# Acupuncture May Be a Potential Complementary Therapy for Alzheimer's Disease: A Network Meta-Analysis

**DOI:** 10.1155/2022/6970751

**Published:** 2022-11-23

**Authors:** Wenshan Yin, Yihan Chen, Anping Xu, Yinshan Tang, Qingtao Zeng, Xin Wang, Zhigang Li

**Affiliations:** ^1^School of Acupuncture-Moxibustion and Tuina, Beijing University of Chinese Medicine, Beijing, China; ^2^Second Clinical Medical College, Beijing University of Chinese Medicine, Beijing, China; ^3^Department of Rehabilitation and Traditional Chinese Medicine, The Second Affiliated Hospital of Zhejiang University School of Medicine, Hangzhou, China; ^4^Information Engineering Institute, Beijing Institute of Graphic Communication, Beijing, China; ^5^Beijing Hospital of Traditional Chinese Medicine, Capital Medical University, Beijing, China

## Abstract

With Alzheimer's disease (AD) becoming a worldwide problem, traditional Chinese medicine (TCM), especially acupuncture, stands out as a complementary therapy because of its feature—“treatment based on syndrome differentiation”. This systematic review and network meta-analysis (NMA) confirms the complement effect of acupuncture and explores the best combination of therapy for AD based on the total effect and activity of daily living scale (ADL). We searched relevant randomized controlled trials (RCTs) that applied acupuncture for treating AD. 58 studies with 4334 patients were included in accordance with PRISMA guidelines. The results showed that for the total effect, the order of probability for the effect: acupuncture + western medicine > acupuncture + herbal medicine > acupuncture > acupuncture + western medicine + herbal medicine. For the ADL score, the order of probability for the effect: acupuncture + western medicine > acupuncture > acupuncture + western medicine + herbal medicine > acupuncture + herbal medicine. The combination of acupuncture and medicine has a better clinical effect than acupuncture only in a way. Acupuncture + western medicine has an obvious and exact improvement in the curative effect from both total effect and ADL score, but further higher quality studies, which can detail the classification of these interventions, are still needed to verify it.

## 1. Introduction

Alzheimer's disease (AD), also known as senile dementia, is a common degenerative disease of the central nervous system in the elderly. AD is the most common type of dementia (accounting for 60% to 80% of all dementia types) [1], mainly manifested as memory impairment, aphasia, apraxia, ignorance, executive dysfunction, as well as personality and behavior changes. With the progression of the disease, patients' abilities in cognition, behavior, and other aspects can gradually decline. Their living quality can be much lower and they eventually lose their cognition and self-care abilities. As the aging of the world's population intensifies, the incidence rate of AD has also increased. As of 2017, the prevalence rate of AD in China was 7.5%; the prevalence rate for people over 80 years old was about 30% [2]. From the current situation of clinical treatment, the cure rate of AD is low. Recently, the age of onset has also been getting younger, and the pathogeny of AD has become much more complicated. AD has gradually become a worldwide problem [3].

At present, western medicine is considered the mainstream treatment for AD. Western medicine mainly uses drugs with functions of inhibiting *β*-amyloid deposition, inhibiting neurofibrillary tangles, increasing cholinergic nerve function, and excitatory neurotransmitters to treat AD [4], such as nimodipine, donepezil hydrochloride, Oracetam, Carbalatin, etc. Because of the complex pathogenesis of AD, drug therapy has its limitations. Drug therapy can only target certain pathogenesis to treat AD, which is deficient in comprehensive treatment. Therefore, various complementary therapies have been developed recently. Traditional Chinese medicine (TCM) therapies, such as herbal medicine and acupuncture, are complementary treatments with huge development potential that have been proven to be effective. They all follow the principle of “treatment based on syndrome differentiation”, which means that clinicians can adjust their selection of herbal medicine or acupoint based on the specific body condition of patients to get a better overall effect. Moreover, previous studies have confirmed that acupuncture has the features of multiple targeting therapy. Moreover, its function of holistic regulation plays an important role in the preventive treatment of AD [5]. The combination of TCM therapies and western mainstream medicine has been constantly innovated and developed, among which the combination of acupuncture and medicine accounts for a certain proportion, and the clinical efficacy of this combination has also been confirmed. However, due to the variation and differences in the prescription of TCM and the selection of acupoints, the clinical efficacy of combined interventions is greatly affected by specific intervention plans. The various intervention plans with great differences in clinical randomized controlled trials (RCTs) may affect the comprehensiveness of therapeutic evaluation because of the limitations on sample size. Clinical studies with large sample sizes are needed to provide evidence for comparing the clinical efficacy of various combinations of acupuncture and medicine for treating AD, to help determine the best combination, and to explore whether acupuncture can complement the mainstream drugs for AD, and provide a reference for the clinical treatment of AD.

Network meta-analysis (NMA) can aggregate data from multiple studies and remedy the limitations on the sample size, allowing us to compare and analyze the clinical efficacy of different interventions for AD based on the network relationships of multiple trials. This study compares and ranks the clinical efficacy of different combination interventions for treating AD (acupuncture + western medicine, acupuncture + herbal medicine, acupuncture, acupuncture + western medicine + herbal medicine, western medicine + herbal medicine, and western medicine) based on NMA to provide more intuitive data evidence for the comparison and application of various combinations of acupuncture and medicine in the clinical treatment of AD.

## 2. Methods

### 2.1. Search Strategy

Two researchers searched PubMed, Embase, Cochrane Library, CBM, CNKI, WanFang Data, and CQVIP databases until August 21, 2021, independently. There were no date limits regarding the publication date of the included studies. In addition, the references of the included studies were traced to obtain other relevant studies to supplement the included studies.

The search was carried out by combining subject terms and free words. All RCTs of acupuncture for treating Alzheimer's disease were collected. We searched for articles in both Chinese and English for more comprehensive materials. Search terms: “Alzheimer”, “Alzheimer's”, “Alzheimer disease”, “AD”, “ATD”, “senile dementia”, “Alzheimer type dementia”, “Alzheimer-type dementia”, “degenerative Alzheimer's disease”, “Alzheimer syndrome”, “presenile dementia”, “Alzheimer sclerosis”, “Acupunctural”, “Acupuncture”, “Acupuncture therapy”, “Acupuncture treatment”, “Scalp acupuncture”, “Needle”, “warm needle”, “temperature needle”, “auricular point sticking”, “auricular acupuncture”, “Fire-needle acupuncture”, “needle warming therapy”, etc. Taking PubMed as an example, the retrieval strategy is shown in Table 1.

### 2.2. Inclusion and Exclusion Criteria

Inclusion and exclusion criteria were formulated based on the principle of PICOS (P-population; I-intervention;C-comparison;O-outcome;S-study design):

The inclusion criteria were as follows: ① Study design: published RCTs. The language of materials was limited to Chinese or English. ② Population: patients who were diagnosed with AD or met the diagnostic criteria, such as “the Diagnostic and Statistical Manual of Mental Disorders, Revised Fourth Edition” (DSM-IVR) [6], which was published by the American Psychiatric Association. There was no limitation in patients' gender, age, nationality, race, occupation, education level, course, and severity of the disease. The baseline of the same RCT was balanced (*P* > 0.05). The participants were allowed to suffer from hypertension, diabetes, hyperlipidemia, and other underlying diseases. ③ Intervention and comparison: The experimental group was treated with various acupuncture methods alone or combined with herbal or western medicine, such as electroacupuncture combined with donepezil hydrochloride, acupuncture combined with Yizhi Jiannao granule, and so on. The control group was treated with herbal medicine, western medicine, or a combination of herbal medicine and western medicine. ④ Outcome: Total effect: According to “Criteria of diagnosis and therapeutic effect of internal diseases and syndromes in traditional Chinese medicine” (issued by the National Administration of Traditional Chinese Medicine), the curative effect can be divided into “cured” (all symptoms disappear), “improved” (symptoms are relieved), and “ineffective” (aggravation or no change in symptoms). “Cured” and “improved” were regarded as effective. The total effect = (the number of “cured” and “improved”/the total sample size)^*∗*^100%; Activity of Daily Living Scale (ADL). Included studies should address one or both of the outcomes mentioned above.

The exclusion criteria were as follows: ① Repeated publications. ② Studies in the diagnosis of vascular dementia. ③ Studies without relative data or unavailable for researchers. ④ Participants had a malignant tumor, diseases of the blood or immune system, mental illness, or other obvious complications. ⑤ The experimental groups or control groups applied other therapies besides acupuncture, herbal medicine, or western medicine, such as doll therapy, hyperbaric oxygen, music-assisted therapy, electric shock therapy, etc. ⑥ The rate of loss to follow-up or drop-off was more than 50%, the data of outcome were missing or wrong obviously, or the efficacy evaluation was unclear.

### 2.3. Study Selection and Data Extraction

Four trained researchers were divided into two groups to screen studies and extract data independently, and another two researchers cross-checked the data. Any disagreement was resolved by discussion. Subsequently, the data were extracted into a unified spreadsheet, and the extraction contents included the following: ① basic information of included studies: title, name, and nationality of the first author, publication year, source of study, fund status, etc.; ② baseline characteristics of objects: sample size of each group, age, course of the disease, etc.; ③ intervention: acupuncture methods (including acupoint selections, reinforcing and reducing techniques, direction of the needle, retaining time of needle, course of treatment, etc.), drug therapeutic schedule; ④ relative information about bias risk assessment: random method, the situation of drop-off and follow-up, etc.; ⑤ outcome: total effect and ADL.

### 2.4. Risk of Bias

Two researchers evaluated the quality of included studies independently according to the bias risk assessment tool, namely ROB 2 [7], recommended by Cochrane5.1.0. Subsequently, the results of the assessment were cross-checked and any disagreement was resolved by a discussion.

The assessment was related to five major domains: ① the randomization process; ② deviations from the intended interventions; ③ missing outcome data; ④ measurement of the outcome; and ⑤ selection of the reported results. The answers to questions involved the five domains were provided as Yes (Y), Probably Yes (PY), Probably No (PN), No (N), or No Information (NI). The whole process of assessment was based on the Cochrane Handbook.

### 2.5. Statistical Analysis

Researchers utilized Stata/SE 16.0 software to construct NMA in a frequentist framework. All the statistical data mentioned below were calculated using Stata/SE 16.0. For dichotomous variables (total effect), odds ratio (OR) was adopted as the effective value. For continuous variables (ADL), mean difference (MD) was adopted as the effective value. The meta-analyses were carried out by calculating the effect values and their 95% credibility interval (CI).

Researchers constructed a network map to depict the comparator arms of various interventions and the relationship between these interventions. Weight the points by the total sample size received for the specific treatment, and weight the lines by the number of researchers, which compared two interventions connected by the line directly.

Researchers calculated the effect values and their standard error (SE) of each research group and constructed a contribution plot to display the contribution of direct and indirect comparison in NMA.

A heterogeneity test was performed through an I^2^ test. Higgins [8] considered that I^2^ was between 0% and 100%. There was no heterogeneity between studies when I^2^ = 0%. The larger the I^2^, the higher the possibility of heterogeneity. It indicated that there was mild heterogeneity when I^2^ = 25%; it indicated that there was moderate heterogeneity when I^2^ = 50%. It indicates a high degree of heterogeneity when I^2^ = 75%. The Cochrane manual believed that when I^2^ > 50%, the research study was considered to be heterogeneous, and a random effects model should be applied. When I^2^ < 50%, the fixed effects model should be applied. If the heterogeneity was high, further subgroup analysis (according to the course of disease and therapy) and meta-regression should be performed to analyze the causes of heterogeneity.

The inconsistency test of each closed loop in the network map was carried out. Researchers calculated the inconsistency factors (IFs), 95% CI, and the heterogeneity parameter *t*^2^ (*t* = Standard deviation<SD>) of each loop to analyze whether there was an inconsistency in each closed loop. The closer the IF gets to 1, the more consistent between different studies. If the lower limit of 95% CI was 1, it meant that the direct comparison results were consistent with the indirect comparison results.

Researchers set “Western medicine” as the original control intervention. We construct an interval prediction graph and an inverted triangle diagram to display the direct and indirect comparison results of different interventions. Treatment ranking was related to the area under the curve. The larger the area, the better the effect of the intervention [9].

A comparison correction funnel plot was applied to analyze whether there was a small sample effect between the studies and to assess the publication bias.

Researchers summarized the selection and usage frequency of acupoint and drugs used in the included studies.

## 3. Results

### 3.1. Results of the Search Process

**Figure 1:**
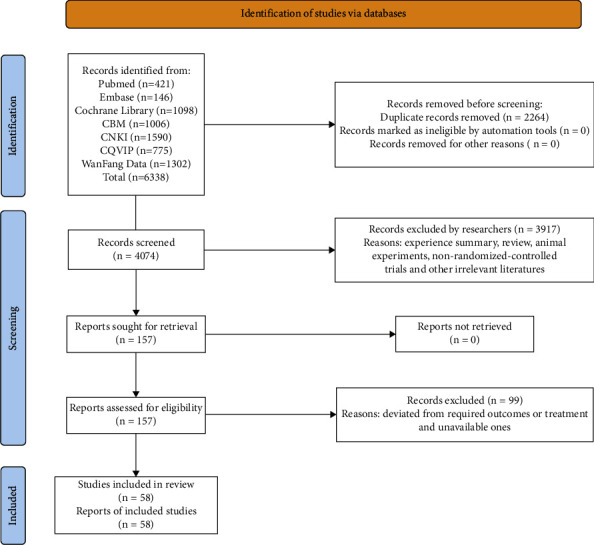
Search process depicted by the PRISMA flowchart.

The total number of obtained records was 6338, including 421 for PubMed, 146 for Embase, 1098 for the Cochrane Library, 1006 for CBM, 1590 for CNKI, 775 for CQVIP, and 1302 for WanFang Data. Records were imported into NoteExpress 3.2.0; then 4074 records after duplicates removed were obtained. Four researchers simply screened titles and abstracts. 157 records were left after excluding experience summary, reviews, animal experiments, nonrandomized controlled trials, and other irrelevant literature. The remaining full-text articles were further screened, and 58 records were left after excluding those that deviated from required outcomes or treatment, as well as unavailable ones. Ultimately, 58 RCTs [10–30] [31–45] [46–67] were included in our research, and the process is depicted in Figure 1.

### 3.2. Characteristics of the Included Studies

As demonstrated in Table 2, 58 articles were included in the research, and 4334 AD patients were recruited in the trial, 2190 for experimental groups and 2144 for comparator groups, respectively. Two studies [40, 64] collected outcomes, respectively, at different stages of treatment. Researchers split these two studies according to the course of treatment into five independent studies. Ultimately, 60 studies were included in the final statistical analysis, with 4542 patients. The total effect and ADL were the main outcomes. 54 studies reported total effects, and 25 studies reported ADL. 7 interventions were included, A-herbal medicine; B-western medicine; C-acupuncture;D-acupuncture + herbal medicine; E-acupuncture + herbal medicine + western medicine; F-acupuncture + western medicine; G-herbal medicine + western medicine.

### 3.3. Risk of Bias and Certainty of Evidence

**Figure 2:**
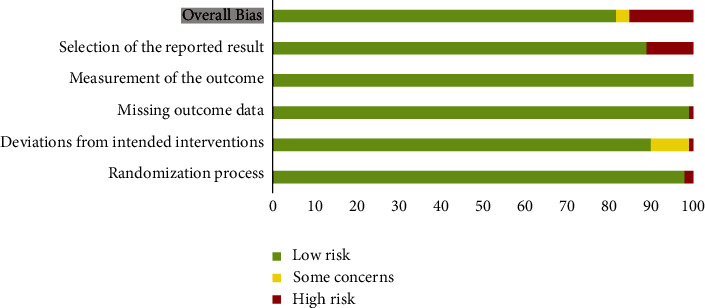
Summary of bias risks based on ROB 2. Over 80% of the included study were assessed as low risk overall.

Researchers used the bias risk assessment tool, named ROB 2, recommended by Cochrane 5.1.0. A total of 5 aspects of the original study were assessed, including the randomization process, deviation from intended interventions, missing outcome data, measurement of the outcome, and selection of the reported result. Included studies were classified as high quality, low quality, or unknown risk bias. The result is depicted in Figure 2 and Table 3.

### 3.4. Total Effect

#### 3.4.1. Network Structure

**Figure 3:**
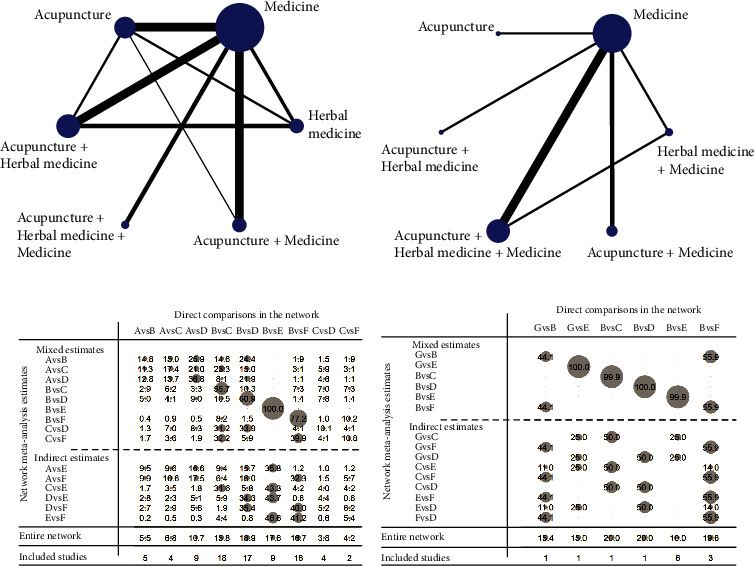
Network diagram comparing treatment outcomes of AD for total effect (a) and ADL (b). The diameter of each dot represents the proportional total weight of all trials in the network that investigated that intervention. The thickness of each line connecting 2 interventions is proportional to the number of trials that investigated that pair of interventions. Contribution plots for treatments of AD for total effect(c) and ADL (d). A-herbal medicine. B-western medicine. C-acupuncture. D-acupuncture + herbal medicine. E-acupuncture + herbal medicine + western medicine. F-acupuncture + western medicine. G-herbal medicine + western medicine. The size of each circle is proportional to the weight attached to each direct or indirect summary effect. The numbers re-express the weights as percentages.

A total of 59 studies reported the total effect, involving 129 arms and 4,414 patients.  Figure 3 depicts the comparative relationship between different interventions. The dots represent the total number of samples in all studies using this intervention. The lines represent the amount of research evidence that directly compared the two interventions connected. An indirect comparative analysis was carried out based on a network structure for two unconnected interventions. The studies involved included six kinds of interventions: herbal medicine, western medicine, acupuncture, acupuncture + herbal medicine, acupuncture + western medicine, and acupuncture + western medicine + herbal medicine. five closed loops have been formed in the network structure (“herbal medicine, acupuncture, acupuncture +  herbal medicine”, “herbal medicine, western medicine, acupuncture”, “western medicine, acupuncture, acupuncture +  herbal medicine”, “western medicine, acupuncture, acupuncture + western medicine”, “herbal medicine, western medicine, acupuncture + herbal medicine”), to provide direct and indirect comparative evidence for NMA.

#### 3.4.2. Contribution Plot

Figure 3 displays the contribution of each direct comparison result to the comprehensive comparison results of NMA, based on the total effect. “Direct comparisons in the network” refers to the direct comparison evidence included in studies. “Mixed estimates” represent comparisons that combine direct and indirect comparison evidence. “Indirect estimates” represent comparisons that are only based on indirect comparison evidence. For example, 25.9 means that the contribution rate of the direct comparison between intervention A (Herbal medicine) and intervention D (Acupuncture + Herbal medicine) for comparing the efficacy of intervention A (Chinese medicine) and intervention B (Western medicine) is 25.9%.

#### 3.4.3. Testing for Heterogeneity and Inconsistency

According to the results of the heterogeneity test, I^2^ = 16.4% < 25%, *P* < 0.05, regarded as low heterogeneity. NMA was carried out under the fixed effects model; applied the inconsistency model was used for NMA in advance, *P*=0.0946 > 0.05. According to the inconsistency test for the closed loop, *P* > 0.05 for each closed loop (Table 4), which indicates no inconsistency among the groups. The consistency model was selected for NMA.

#### 3.4.4. Network Meta-Analysis

**Figure 4:**
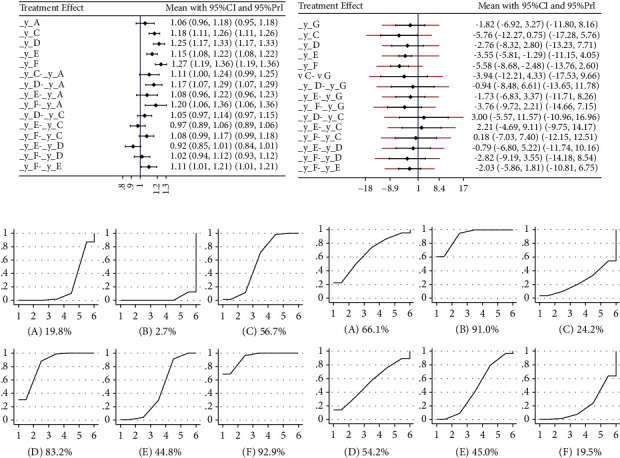
Forest plot of treatment differences on the standard normal scale for total effect (a) and ADL (b). The ineffectiveness line (vertical line, X = 1 or X = 0) means an equal ratio. Each horizontal line connects the upper and lower limits of the 95% confidence interval for study, and the length of lines indicates the range of the confidence interval. If the line crossed X = 1 or X = 0, the study was not statistically significant. If the line totally falls on the left side of X = 1 or X = 0 means worse efficacy and the right side for the opposite. The diamond-shaped blocks are the locations corresponding to the OR values. Surface under the cumulative ranking curves for all interventions for total effect (c) and ADL (d). A-Herbal medicine. B-Western medicine. C-Acupuncture. D-Acupuncture + Herbal medicine. E-Acupuncture + Herbal medicine + Western medicine. F-Acupuncture + Western medicine. G-Herbal medicine + Western medicine. Y axis represents cumulative probability and X axis represents rank. Comparing the cumulative probability of the same control ranking, the higher ranking (6⟶1) with a higher cumulative probability means a better curative effect.

Figure 4 displays the results of direct and indirect comparisons; _y_A, _y_C, _y_D, _y_E, and _y_F represent comparison results between interventions A, C, D, E, F, and intervention B, respectively. Labels, like_y_C-_y_A, _y_D-_y_A, etc., represent the comparison results between the two interventions mentioned. The results indicate that the curative effects of acupuncture, acupuncture + herbal medicine, acupuncture + western medicine, and acupuncture + herbal medicine + western medicine are better than that of western medicine. The curative effects of acupuncture, acupuncture + herbal medicine, and acupuncture + western medicine are better than those of herbal medicine. The curative effect of acupuncture + western medicine is better than acupuncture + herbal medicine + western medicine. The differences in the remaining comparisons were not statistically significant.

The results mentioned above can also be obtained from the inverted triangle diagram (Table 5). The 95% CI must not contain 1; otherwise, the differences in comparisons are not statistically significant. If the OR value is greater than 1, it means that the interventions sorted by the column have better efficacy than the interventions sorted by the line.

The surface under the cumulative ranking curve (SUCRA) (Figure 4) shows that the combination of acupuncture and western medicine is the most effective intervention for treatment. The order of probability for the effect: acupuncture + western medicine > acupuncture + herbal medicine > acupuncture > acupuncture + western medicine + herbal medicine > herbal medicine > western medicine. the order of the curative effect of the intervention combined with acupuncture: acupuncture + western medicine > acupuncture + herbal medicine > acupuncture > acupuncture + western medicine + herbal medicine.

#### 3.4.5. Small Sample Effect and Bias

**Figure 5:**
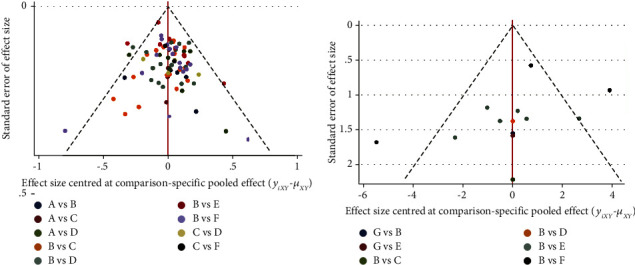
Funnel plot assessment of publication bias for total effect (a) and ADL (b). A-Herbal medicine. B-Western medicine. C-Acupuncture. D-Acupuncture + Herbal medicine. E-Acupuncture + Herbal medicine + Western medicine. F-Acupuncture + Western medicine. G-Herbal medicine + Western medicine. Most of the dots are symmetrically distributed on both sides of the vertical line of the X = 0, indicating a low possibility of both bias and the small sample effect.

The comparison-correction funnel plot (Figure 5) displays that the dots are slightly asymmetrically distributed on both sides of the vertical line of the X = 0. Five studies, including “western medicine” vs “acupuncture + herbal medicine + western medicine”, “western medicine” vs “acupuncture + herbal medicine”, “herbal medicine” vs “acupuncture + herbal medicine”, “western medicine” vs “acupuncture + western medicine” are from the line of 95% CI in Figure 5, which shows that asymmetry may be caused by heterogeneity.

### 3.5. ADL Score

#### 3.5.1. Heterogeneity Test and Subgroup Analysis

According to the results of the heterogeneity test, I^2^ = 94.4% > 75%, *P* < 0.05, regarded as high heterogeneity. Researchers performed a subgroup analysis of the included materials according to the course of AD (Studies were divided into 4 subgroups: less than 1 year, 1–3 years, 3–5 years, and 5–10 years). I^2^ of groups “less than 1 year” and “5–10 years” decreased to 80.6% and 80.3%, respectively, and the I^2^ of the remaining groups did not change significantly. Researchers performed a meta-regression based on the course of AD and interventions, but the heterogeneity remained unchanged. Moreover, there was no reason for heterogeneity was found. Since the number of studies in the group “less than 1 year” is too small (2 studies in total) and the groups “1–3 years” and “3–5 years” have high heterogeneity (>90%), only the “5–10 years” group was involved in NMA. NMA was carried out under the random effects model.

#### 3.5.2. Network Structure

A total of 11 studies were involved in the “5–10 years” group, involving 23 arms and 842 patients.  Figure 3 depicts the comparative relationship between different interventions. The dots represent the total number of samples in all studies using this treatment. The lines represent the amount of research evidence that directly compared the two treatments connected. An indirect comparative analysis was carried out based on the network structure of two unconnected interventions. The studies involved included six kinds of interventions: herbal medicine + western medicine, western medicine, acupuncture, acupuncture + herbal medicine, acupuncture + western medicine, and acupuncture + western medicine + herbal medicine. One closed loop has been formed in the network structure (“herbal medicine + western medicine–acupuncture—acupuncture + herbal medicine + western medicine”) to provide direct and indirect comparative evidence for NMA.

#### 3.5.3. Contribution Plot

Figure 3 displays the contribution of each direct comparison result to the comprehensive comparison results of NMA, based on the ADL score. For example, 44.1 means that the contribution rate of the direct comparison between herbal medicine + western medicine and acupuncture for comparing the efficacy of these two interventions is 44.1%.

#### 3.5.4. Testing for Inconsistency

Applied the inconsistency model for NMA in advance, *P*=0.4132 > 0.05. According to the inconsistency test for closed loop, *P*=0.279 > 0.05 (Table 6), which indicated no inconsistency among the groups. A consistency model was selected for NMA.

#### 3.5.5. Network Meta-Analysis

Figure 4 displays the results of direct and indirect comparisons. The results indicate that the curative effects of acupuncture + western medicine and acupuncture + herbal medicine + western medicine are better than those of Western medicine. The differences in the remaining comparisons were not statistically significant. The results mentioned above can also be obtained from the inverted triangle diagram (Table 7).

The SUCRA (Figure 4) shows that Western medicine is the most effective intervention for treatment. The order of probability for the effect: acupuncture + western medicine > acupuncture > acupuncture + herbal medicine + western medicine > acupuncture + herbal medicine > herbal medicine + western medicine > western medicine. The order of the curative effect of the intervention combined with acupuncture: acupuncture + western medicine > acupuncture > acupuncture + western medicine + herbal medicine > acupuncture + herbal medicine.

#### 3.5.6. Small Sample Effect and Bias

The comparison-correction funnel plot (Figure 5) displays that most of the dots are symmetrically distributed on both sides of the vertical line of X = 0, indicating a low possibility of both bias and the small sample effect.

### 3.6. Usage of Acupoints and Drugs

**Figure 6:**
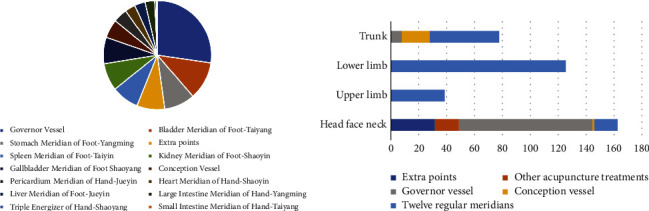
Proportion of meridians chosen by studies included (a). The pie chart area displays application frequency of meridians. Most studies selected Governor the vessel, while few studies select three hand-yin meridians or three hand-yang meridians. Proportion of body parts chosen by studies included (b). The chart shows maximum amount and most abundant meridians of head, face and neck acupoints and opposite for upper limb.

**Figure 7:**
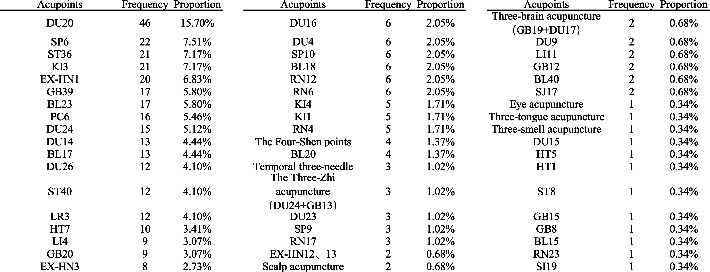
Frequency rank of acupoints chosen by studies included. The head, neck, and lower limb acupoints were more selected, which correspond to the local and distal acupoint selection.

Most studies selected the Governor vessel, three foot-yang meridians, extra acupoints, and three foot-yin meridians, with few acupoints selected for three hand-yin meridians and three hand-yang meridians relatively. Compared with other parts of the body, the acupoints on the head, face, and neck, including Governor vessel acupoints, extra acupoints, other acupuncture treatment methods (including the four-shen acupuncture, temporal three-needle, the three-zhi Acupuncture, etc.), acupoints of twelve regular meridians and conception vessel acupoints, were chosen mostly among the 57 kinds of literature included. The number of selected lower limb acupoints is the second, including only acupoints of twelve regular meridians (Figure 6). The top ten ranked frequencies of chosen acupoints are DU20, SP6 (confluent acupoint of three foot-yin meridians), ST36 (He-sea point of foot-yangming meridian), KI3 (Shu-stream acupoints of foot-shaoyin meridian), EX- HN1, GB39 (marrow convergence), BL23 (kidney back-shu point), PC6 (connecting point of hand-jueyin meridian), DU24, and DU14 (confluent acupoint of governor vessel, three foot-yang, and hand-yang meridians), most for located acupoints and several for nourishing kidney yin (Figure 7). Herbal medicine of the studies included was mainly for tonifying the spleen and kidney by activating blood circulation to dissipate stasis, while donepezil was mostly for western medicine.

### 3.7. Adverse Events

Eleven included studies reported the presence of adverse events(Table 8). Due to the limited number of included studies that reported adverse events, it was not analyzed using NMA.

## 4. Discussion

AD is a common degenerative disease of the central nervous system in the elderly, whose pathogeny is complex and difficult to be explained. There are many interventions for AD used in clinical settings, such as drug therapy, acupuncture, music therapy, exercise therapy, memory therapy, and so on. Acupuncture has the functions of restoring consciousness and resuscitation, promoting blood circulation, replenishing qi and regulating blood, and replenishing the spleen and kidney [68]. In addition, acupoints can be selected flexibly according to the specific body condition of the patient, to improve the patient's symptoms and overall physical condition in a targeted manner. Also, because of its small side effects and good tolerance [69], acupuncture is widely applied for treating AD. The combined application of acupuncture and medicine (herbal medicine or western medicine) has gradually increased recently, and its efficacy has also been confirmed by clinical research studies.

Researchers searched for relative studies and utilized NMA to evaluate the curative effect of acupuncture and the combined treatment of acupuncture and medicine based on the total effect and ADL score. For the total effect, the curative effects of acupuncture, acupuncture + herbal medicine, acupuncture + western medicine, and acupuncture + herbal medicine + western medicine are all better than those of western medicine. The curative effects of acupuncture, acupuncture + herbal medicine, and acupuncture + western medicine are better than those of Herbal medicine. The curative effect of acupuncture + western medicine is better than acupuncture + herbal medicine + western medicine. The differences in the remaining comparisons were not statistically significant. For the ADL score, the curative effects of acupuncture + western medicine and acupuncture + herbal medicine + western medicine are better than those of western medicine. The differences in the remaining comparisons were not statistically significant. The SUCRA shows that the top two interventions that have the best efficacy for total effect are acupuncture + western and acupuncture + herbal medicine (acupuncture + western > acupuncture + herbal medicine). The top two interventions that have the best efficacy for the ADL score are acupuncture + western medicine and acupuncture (acupuncture + western medicine > acupuncture). Results show that acupuncture combined with medicine has a better clinical effect than acupuncture for treating AD. Acupuncture + herbal medicine is more effective for improving the total effect, but there are certain disadvantages in improving the ADL score. The combination of acupuncture and western medicine has an impressive effect on both the total effect and the ADL score.

Acupuncture + herbal medicine and acupuncture + western medicine both have impressive effects on improving the total effect, but they both have worse effects than only applying acupuncture when adding a variety of medicine (applying acupuncture + herbal medicine + western medicine). Acupuncture + herbal medicine has an impressive effect on improving the total effect, but it has a worse effect on improving ADL scores. Researchers speculated that the reason for this contradiction in the sorting of curative effects may be related to the signaling pathways that various treatments affect the body. The most commonly used herbal medicines for treating AD, such as *Salvia miltiorrhiza*, *Ligusticum chuanxiong*, Noto ginseng, turmeric, *Herba epimedium*, and so on, can treat AD by inhibiting the formation and deposition of amyloid *β*-protein (A*β*), inhibiting the hyperphosphorylation of the protein tau, antagonizing oxidative stress damage and neuronal apoptosis, or playing an anti-inflammatory effect, etc [70–74]. In particular, herbal medicine for removing blood stasis is closely related to the body's autophagy, which can enhance autophagy and regulate the content of A*β* and protein tau [75]. The most commonly used Western medicine mentioned in the included studies, such as nimodipine, donepezil, and so on, mostly focus on improving symptoms and have a therapeutic effect on AD by inhibiting cholinesterase, regulating the concentration of calcium ions in the brain, and protecting the structure of neurons [9, 76–78]. Acupuncture can promote autophagy at different levels to treat AD by stimulating specific acupoints. The regulating function of acupuncture on autophagy is bidirectional, which can not only promote but also inhibit autophagy. Acupuncture can also adjust the body's oxidative defense system and reduce the toxic effects of excessive free radicals on the nervous system [79, 80]. The mechanisms of acupuncture, herbal medicine, and western medicine for treating AD have their specific parts and similar parts. There is saturation in signal transduction and various physiological processes. Once the signal stimulation of the same pathway reaches saturation, it may have no obvious enhancement of the effect, even produce a degenerative effect. Therefore, the combined application of acupuncture and herbal or western medicine may produce different comprehensive effects due to the compatibility of herbal medicines or acupoints. A combination of multiple medicines may also lead to differences in efficacy. Further research is still needed to verify it.

The results of the usage of acupoints show that the selection of acupoints for treating AD is diverse and complex and distributes in various parts of the body, but all of them have a therapeutic effect on AD indeed. It reflects the treatment principles of combining the main symptoms and concurrent syndromes, treating based on syndrome differentiation, and selecting acupoints based on syndromes. [81] Although compared with western medicine, acupuncture has poor function targeting treating AD, the principle of acupoint selection based on syndrome differentiation and the multidirectional effect of acupuncture makes it not only have the effect of treating AD but also regulates the whole body condition. This may be the reason why the combination of acupuncture and medicine is better than western medicine alone.

A total of 11 included studies mentioned adverse events after treatment. Adverse events mentioned the most frequently were reactions of the digestive system (nausea, vomiting, abdominal distension, diarrhea, loss of appetite) and the central nervous system (dizziness, insomnia). Although the occurrence of adverse events is affected by the patient's age, gender, and other factors [82], interventions must have a certain relationship with the adverse events. We analyzed the types of interventions used in studies with adverse events. We found that the proportion of Western medicines was the highest (81.8%). Among the included studies, donepezil was the western medicine used the most frequently. Studies have shown that adverse events to the digestive system and central nervous system are the most common adverse events of donepezil [83], which is consistent with the adverse events reported in the included studies to some extent. The mechanism of donepezil's adverse reaction may be related to its inhibition of cholinesterase. It can lead to excessive cholinergic action and cause nausea, vomiting, abdominal distension, and other gastrointestinal reactions, or cause a disorder of neurotransmitters in the central nervous system and cause dizziness, insomnia, and other reactions. [82] Among the 11 studies, 3 studies reported similar adverse events under the combined treatment of acupuncture and herbal medicine. Therefore, we believe that although the occurrence of adverse events is closely related to the use of western medicine, it is also affected by acupuncture and herbal medicine.

In the included studies, acupoints on the head were mostly used for treating AD, whereas acupoints correlated with gastrointestinal function were rarely used. Based on the brain-gut axis theory, some active peptides and neurotransmitters exist in both the brain and the gastrointestinal tract. The gastrointestinal function is closely related to the brain function and can interact with each other. [84] Moreover, studies have shown that the dysregulation of intestinal flora may also lead to AD. [85, 86] Due to the high frequency of gastrointestinal reactions in adverse reactions, researchers believe that RN12, BL21, and other acupoints correlated with gastrointestinal function can be appropriately selected in clinical practice to supplement the therapeutic effect of acupoints on the head and to prevent and alleviate adverse events. The original material on adverse events is not adequate enough, so the conclusion about adverse events should be considered comprehensively and carefully used.

## 5. Limitations

This study has several limitations in the following areas. First, the included studies are mostly small sample studies, and the original data of ADL have high heterogeneity, which may affect the research statistically. More databases need to be searched to increase the number of studies included in the analysis. Second, on account of that western medicine, herbal medicine, and herbal medicine + western medicine were only used as control groups in this study, RCTs, which did not use acupuncture as a study group, but only used western or herbal medicine as study groups, were not included. Therefore, the relative curative effect rankings of western medicine, herbal medicine, and herbal medicine + western medicine are less of the reference value. Third, interventions in the included studies were only classified into acupuncture, acupuncture + herbal medicine, acupuncture + western medicine, and acupuncture + herbal medicine + western medicine. The therapeutic evaluation might be influenced due to classification, which is not detailed enough. Fourth, the current study compares the efficacy of acupuncture and combined therapy of acupuncture and medicine in the treatment of AD and the complementary effect of acupuncture on drug therapy. Therefore, other treatments for AD, such as music therapy and doll therapy, have not been involved in this study. Subsequent studies can expand the research scope and compare the efficacy of various treatments for AD comprehensively. Fifth, more scales can be included in the analysis to assess the efficacy of interventions more completely.

## 6. Conclusions

In conclusion, the combination of acupuncture and medicine has a better clinical effect than acupuncture in a way. Acupuncture + western medicine has an obvious and exact improvement in the curative effect from both the total effect and ADL score. Therefore, the researchers believe that the development of the combination therapy of acupuncture and medicine is advantageous and reasonable for treating AD and that acupuncture does have a complementary effect on drug therapy. It has prompted clinicians to practice combination therapy of acupuncture and medicine and use the principle of selecting acupoints based on syndrome differentiation flexibly to improve the therapeutic effect of AD. Acupuncture should be used appropriately to prevent and alleviate the adverse reactions that may occur during the treatment of AD. Researchers can study and compare the clinical efficacy of different combinations of acupuncture and medicine on patients with different syndromes to determine the combinations that can clearly reduce or improve clinical efficacy and to refine the selection of specific acupoints or methods of acupuncture and prescriptions, and to provide more accurate guidance for the clinical treatment of AD.

## Figures and Tables

**Table 1:** PubMed search strategy.

#1	Alzheimer disease[MeSH]

#2	Alzheimer[Tiab] OR Alzheimer's[Tiab] OR Alzheimer disease[Tiab] OR AD[Tiab] OR ATD[Tiab] OR Senile dementia[Tiab] OR Alzheimer type dementia[Tiab] OR Alzheimer-type dementia[Tiab] OR Degenerative Alzheimer's disease[Tiab] OR Alzheimer syndrome[Tiab] OR Presenile dementia[Tiab] OR Alzheimer sclerosis[Tiab]

#3	#1 OR #2

#4	Acupuncture[MeSH] OR Acupuncture therapy[MeSH]

#5	Acupunctural[Tiab] OR Acupuncture[Tiab] OR Acupuncture therapy[Tiab] OR Acupuncture Treatment[Tiab] OR Scalp Acupuncture[Tiab] OR Needle[Tiab] OR warm Needle[Tiab] OR Temperature needle[Tiab] OR Auricular point sticking[Tiab] OR Auricular Acupuncture[Tiab] OR Fire-needle Acupuncture[Tiab] OR Needle warming Therapy[Tiab]

#6	#4 OR #5

#7	#3 AND #6

**Table 2:** Baseline characteristics included in NMA of the treatment of AD patients.

Study ID	*Group*		Age (mean ± SD)	Course (Month-M; Year-Y)	Follow times/d	*Interventions*		Outcome	Acupoints
	Exp	Com				Exp	Com		
Lei [48]	22	20	Average 67.3/66.4	Average 19.2/17.8M	28	D	B	Total effect	EX-HN1, DU20, DU24, HT7, PC6, LI4, SP6, ST36, KI3
Li et al. [28]	35	18	67 ± 4/66 ± 4	3.1 ± 1.1/3.0 ± 1.3y	None	D	A	Total effect, ADL	DU20/EX-HN1, GB20, BL23
	37	14	65 ± 6/65 ± 7	3.0 ± 1.6/2.7 ± 2.0y		C	B		
He et al. [43]	32	30	55–69/50–69	None	None	F	B	Total effect	DU20
Ou et al. [23]	16	14	65.53 ± 6.8/64.72 ± 7.6	2.9 ± 1.6/2.7 ± 2.0y	56	C	B	Total effect, ADL	DU20/EX-HN1, BL23
Ou et al.[23]	16	14	65.5 ± 6.8/64.7 ± 3.4	2.9 ± 1.6/2.7 ± 2.0y	56	C	B	Total effect	DU20/EX-HN1, BL23
Xia [38]	30	30	67.93 ± 4.68/16.40 ± 4.26	67.70 ± 5.49/16.03 ± 4.00M	7	D	B	Total effect, ADL	DU20, EX-HN1, DU24, HT7, KI3, ST36, GB39, KI4, BL23, SP6
He [55]	30	30	63.83 ± 6.24/66.97 ± 7.34	17.13 ± 4.44/18.93 ± 5.05M	28	F	B	Total effect, ADL	DU20, DU14, DU4, DU9
Zhou [67]	50	50	71.30 ± 8.20/68.60 ± 10.10	1.80 ± 0.50/1.70 ± 0.20y	28	D	B	Total effect, ADL	EX-HN3, DU20, DU23, KI3, GB39, GB20, LR3, BL18, LI4, BL23, PC6, BL17
Chen et al. [19]	51	51	68.59 ± 4.36/68.59 ± 4.36	2.45 ± 0.71/2.45 ± 0.71y	28	C	B	Total effect, ADL	DU20, DU24, GB20, SP10, RN17, RN12, GB12, ST36, RN6
Li et al. [11]	45	45	(70.52 ± 5.34)/(70.56 ± 5.32)	(5.46 ± 1.39)/(5.42 ± 1.35)y	90	E	B	Total effect, ADL	DU26, PC6, SP6, GB20, DU20, HT7, EX-HN1, EX-HN3
Chen et al.[25]	40	40	69.5 ± 10.3/70.1 ± 9.6	6.1 ± 2.9/5.7 ± 3.3y	28	E	B	ADL	EX-HN3, EX-HN1, DU20, DU24, GB20, KI3, GB39, LI4, LR3, BL18, BL23, PC6, BL17
Peng et al. [41]	25	25	69.4 ± 5.4/69.5 ± 5.3	7.5 ± 1.8/7.6 ± 1.7y	10	F	B	Total effect	DU24, DU20, DU14, DU16, DU4, KI1
Guan [18]	30	30/30	70.5 ± 9.3/70.2 ± 9.5/69.3 ± 10.2	5.7 ± 3.2/5.6 ± 3.5/6.0 ± 6.0y	7	E	B/*G*	ADL	Scalp acupuncture, BL23, GB39, KI3, ST36, DU26
Wang and Wang [53]	45	45	68.89 ± 3.22/69.18 ± 3.17	8.59 ± 2.02/8.93 ± 2.17Y	28	E	B	Total effect, ADL	DU20, DU24, DU16, DU4
Ni [66]	33	32	73.25 ± 2.70/74.14 ± 2.76	3.90 ± 1.52/3.20 ± 1.30y	28	D	D	Total effect	DU20, DU14
Chen [49]	31	31	48–70/50–73	6M-5y/5m-7y	90	E	B	Total effect	The four-shen points, Three-brain acupuncture, The three-zhi acupuncture, Temporal three-needle
Jia et al. [15]	41	41	5.11 ± 6.53/74.50 ± 6.83	2.42 ± 1.00/2.50 ± 1.02Y	84	C	B	Total effect, ADL	RN17, RN12, RN6, SP10
Tian and Cheng [17]	35	35	50–80	None	5/30	F	B	Total effect	EX-HN1, DU20, DU24, EX-HN3, DU26, DU16, HT7
Geng [10]	36	36	68–87/69–85	5.19/5.31M	7	C	B	Total effect	DU26, PC6, SP6, KI1, DU24, EX-HN1, DU20, LI4, LR3, GB39
Qing [39]	30	30	70.31 ± 5.43/70.27 ± 5.93	12.63 ± 2.94/12.30 ± 3.09M	7	D	B	Total effect	DU20, EX-HN1, KI3, KI4, GB39, RN6, ST36
Liang [60]	37	37	73.8 ± 6.7/72.5 ± 5.2	7.1 ± 1.8/6.4 ± 1.3Y	14	F	B	Total effect, ADL	DU20, ST36, GB39, EX-HN1, KI4, KI3
Zhang [24]	41	41	72.19 ± 2.61	24.16 ± 3.08 M	20	E	B	Total effect, ADL	DU26, GB39, ST36, BL23, KI3
Wei et al. [26]	33	33	68.37 ± 5.37/68.71 ± 5.77	7.77 ± 1.65/(7.88 ± 1.67Y	None	F	B	ADL	DU20, KI1
Ben et al. [42]	37	37	71.5 ± 4.7/70.2 ± 4.6	3.2 ± 1.9/3.0 ± 1.4y	84	C	B	Total effect	ST36, ST40
Zhu [57]	40	40	70 ± 2/66 ± 2	9.02 ± 0.31/9.13 ± 0.25y	10	C	B	Total effect	DU20, EX-HN1, ST36, KI3, KI4, GB39
Yao et al. [64]	24	24	76.52 ± 6.365/76.43 ± 6.25	3.6 ± 1.65/3.6 ± 1.65Y	30	E	B	Total effect	DU20
Zhang et al. [59]	40	40	average77.6/76.8	average9.5/9.2y	30	E	B	Total effect	DU20, GB39, EX-HN1, LI4, ST36
Lin [35]	30/30	30	69.7 ± 5.36/73.2 ± 4.81/71.6 ± 5.22	55.9 ± 6.18/53.7 ± 5.92/61.3 ± 8.46d	28	C/F	B	Total effect, ADL	The four-shen points, Three-brain acupuncture, The three-zhi acupuncture, Temporal three-needle
Wang et al. [20]	36	36	72.05 ± 3.70/70.31 ± 3.79	3.33 ± 1.98/2.60 ± 1.51y	84	C	B	Total effect	DU20, DU14
Liu [52]	20	20	72.2 ± 4.8/74.4 ± 4.7	40.6 ± 13.4/35.7 ± 12.9 M	84	C	B	Total effect	DU20, DU14
	20	20	73.2 ± 4.9/70.7 ± 4.4	36.5 ± 13.7/33.3 ± 12.1 M	84	D	A	Total effect	
Chen et al. [40]	40	40	67.19 ± 10.53/68.32 ± 9.4	5.36 ± 2.84/5.80 ± 3.48Y	20	E	B	ADL	EX-HN3, EX-HN1, DU20, DU24, DU23, GB20, KI3, GB39, LI4, LR3, BL18, BL23, PC6, BL17
Wang and Li [51]	50	50	69.79 ± 6.52/71.47 ± 6.32	5.54 ± 2.25/5.39 ± 2.03y	10/20	F	B	Total effect, ADL	DU26, SP6, PC6, GB20, GB12, EX-HN12, EX-HN13, SJ17
Li [37]	51	51	71.28 ± 2.34/71.25 ± 2.38	4.75 ± 1.33/4.72 ± 1.30y	10	F	B	Total effect, ADL	SP6, ST36, EX-HN1, ST40, HT7, PC6, KI3, DU26, EX-HN3, DU20, GB20, DU16
Wang et al. [63]	27	28	70.7 ± 9.1/70.3 ± 8.0	5.8 ± 0.6/5.0 ± 1.1y	28	F	B	Total effect	None
Ma [58]	30	30	63.83 ± 6.24/66.97 ± 7.34	17.13 ± 4.44/18.93 ± 5.05 M	8	C	B	Total effect, ADL	DU20, DU14, DU4, BL23, GB39, KI3
Jin [30]	26	26	63.73 ± 9.12/64.88 ± 8.97	4.91 ± 2.29/4.86 ± 2.32y	180	F	B	Total effect, ADL	The four-shen points
Chen et al. [44]	50/50	50	73.16 ± 7.69/72.86 ± 7.23/72.06 ± 6.97	4.02 ± 0.11/3.96 ± 0.15/3.84 ± 0.19	90	F/C	B	Total effect	DU20, EX-HN1
Liu [14]	40	40	66.21 ± 3.72/65.65 ± 3.24	3.86 ± 1.23/3.42 ± 1.12y	90	D	A	Total effect	DU20, DU26, PC6, SP6, ST40, KI3
Zhang et al. [46]	30	30	72.36 ± 4.14/72.31 ± 4.12	3.25 ± 1.29/3.18 ± 1.26Y	90	D	B	Total effect	DU20, KI1
Wang et al. [16]	31	31	72.74 ± 8.36/75.77 ± 7.03	2.50(1.00,4.25)/3.00(2.00,5.00)y	56	F	B	Total effect	DU20, EX-HN3, GB15, GB8, GB20, LI4, LI11, ST36, LR3
Xia et al. [56]	30	30	49 ± 11/50 ± 12	3.79 ± 0.27/4.07 ± 0.27y	56	F	B	ADL	DU20, DU16
Zhang [65]	25	25	64.23 ± 1.56/65.42 ± 2.45	4.12 ± 1.42/4.23 ± 1.42y	None	D	B	Total effect	PC6, DU26, DU20, SP6, DU14, HT7
Zhang et al. [34]	46	46	72.6 ± 9.2/71.7 ± 8.7	None	90	F	B	Total effect, ADL	(BL23, BL20, DU20)/(BL15, ST36, EX-HN1)
Wang and Li [27]	60	60	69.5 ± 3.5/69.2 ± 3.6	3.3 ± 0.8/3.5 ± 0.9y	28	E	B	Total effect, ADL	Temporal three-needle, three-brain acupuncture, The four-shen points, The three-zhi acupuncture, HT7
Li et al. [21]	43	43	76.5 ± 6.3/77.5 ± 6.8	3.1 ± 0.8/2.9 ± 0.7y	None	D	A	Total effect, ADL	DU20, DU14, DU24, DU16
Tao and Li [47]	45	45	64.23 ± 1.56/65.42 ± 2.45	4.12 ± 1.42/4.23 ± 1.43y	None	D	B	Total effect	KI1, DU20
Chen et al. [62]	48	48	74.36 ± 5.47/75.13 ± 5.81	3.42 ± 0.73/3.29 ± 0.68Y	90	F	B	Total effect, ADL	DU20, DU26, PC6, SP6, GB39, ST40, KI3
Liu [36]	20	20/20	67.2 ± 4.2/68.3 ± 5.1/68.8 ± 5.6	None	28	D	A/B	Total effect	EX-HN1, DU20, HT7, ST36
Zhang [50]	32	28	51–80	None	15	D	A	Total effect	DU26, PC6, SP6, DU20, DU14, HT7, GB39, Eye acupuncture
Liu et al. [29]	24	22	56–78/55–77	8M-5y	28	D	B	Total effect	EX-HN1, DU20, DU24, HT7, PC6, LI4, SP6, ST36, LR3
Li and Li [33]	40	40	70.24 ± 5.14/69.37 ± 4.67	4.85 ± 1.50/4.82 ± 1.47y	28	D	B	Total effect, ADL	DU20, DU26, DU15, DU24, DU14, DU9
Zhao et al. [45]	16	16	67 ± 2.12	5.17 ± 1.05Y	60	C	B	Total effect, ADL	DU20, DU14
Zhu et al. [12]	20	20	72.3 ± 6	6M-3y	56	C	B	Total effect	DU20, BL23, SP10, BL17
	20	20			56	D	A		
Liu et al. [54]	40	40	69.16 ± 2.12/68.09 ± 6.24	10.05 ± 2.60/9.79 ± 5.22	7	C	B	Total effect	Three-smell acupuncture
Peng and Dong [22]	28	28/28	62–79	1-8Y	84	D	B/A	Total effect, ADL	DU20, EX-HN1, DU14, RN4
Ji et al. [61]	53	53	None	None	30	C	B	Total effect	DU20, PC6
Li et al. [13]	20/20	20	55–80	None	84	C/D	A	Total effect	BL23, BL17, HT7, DU20
Luo et al. [31]	48	48	67.7 ± 7.2	Over 6M	25	C	B	Total effect	DU14, BL23, KI3, ST36

A-herbal medicine; B-western medicine; C-acupuncture. D-acupuncture + herbal medicine; E-acupuncture + herbal medicine + western medicine; F-acupuncture + western medicine; G-herbal medicine + western medicine.

**Table 3:** Quality assessment according to the ROB 2.

Reviewer	*He et al.* [43]		*Zhang* [50]		*Li and Li* [33]		*Chen et al*. [19]		*Geng* [10]		*Liu* [36]		*Xia* [38]		*Tao and Li* [47]		*Ji et al.* [61]		*Li and Li* [33]		*Jia et al.* [15]		*Wang et al.* [20]	
	Assessment	Comments	Assessment	Comments	Assessment	Comments	Assessment	Comments	Assessment	Comments	Assessment	Comments	Assessment	Comments	Assessment	Comments	Assessment	Comments	Assessment	Comments	Assessment	Comments	Assessment	Comments
Selection of the reported result	High	The study did not explain the specific treatment time of each treatment group, and it is suspected that there are possibilities of choosing the results	High	The study did not explain the specific treatment time of each treatment group, and it is suspected that there are possibilities of choosing the results	High	The study did not explain the specific treatment time of each treatment group, and it is suspected that there are possibilities of choosing the results	Low	—	Low	—	High	The study treated adverse events and were suspected of choosing the results	High	The study treated adverse events and were suspected of choosing the results	High	The study treated adverse events and were suspected of choosing the results	Low	—	High	The control group of the study was not treated with specific western medicine for AD, but only treated with conventional western medicine for other diseases.	Low	—	High	The study applied different outcomes from the original outcomes (not the outcomes used in this study).
Measurement of the outcome	Low	—	Low	—	Low	—	Low	—	Low	—	Low	—	Low	—	Low	—	High	The study had an unexplained dropout.	Low	—	Low	—	Low	—
Missing outcome data	Low	—	Low	—	Low	—	Low	—	Low	—	Low	—	Low	—	Low	—	Low	—	Low	—	Low	—	Low	—
Deviations from intended interventions	Low	—	Low	—	Low	—	Some concerns	The study was suspected of including dropout data into the analysis process	Some concerns	The study was suspected of including dropout data into the analysis process	Some concerns	The deviations may not likely to have affected the outcome.	Low	—	Low	—	Low	—	Low	—	Some concerns	The study reduced or stopped the medicine due to the adverse events during the process of treatment.	Low	—
Randomization process	Low	—	Low	—	Low	—	High	The study might take antidepressant and anxiety drugs at the same time in the treatment process and apply random method based on the date of admission.	Low	—	Low	—	Low	—	Low	—	Low	—	Low	—	Low	—	Low	—

**Table 4:** Evaluation of inconsistency using loop-specific heterogeneity estimates for total effect.

Loop	ROR	z_value	*p*_value	CI_95	Loop_Heterog_tau2
A-C-D	1.244	1.622	0.105	(1.00, 1.62)	0.000
A-B-C	1.232	1.641	0.101	(1.00, 1.58)	0.000
B–C-D	1.171	1.652	0.099	(1.00, 1.41)	0.000
B–C–F	1.106	0.994	0.320	(1.00, 1.35)	0.000
A-B-D	1.064	0.522	0.601	(1.00, 1.34)	0.004

A-Herbal medicine. B-Western medicine. C-Acupuncture. D-Acupuncture + Herbal medicine. E-Acupuncture + Herbal medicine + Western medicine. F-Acupuncture + Western medicine.

**Table 5:** Inverted triangle diagram for total effect.

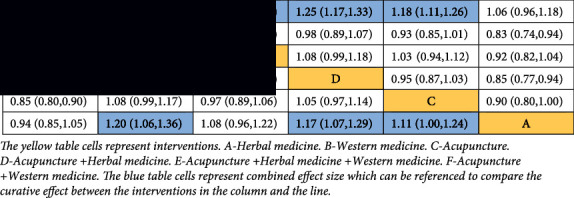

**Table 6:** Evaluation of inconsistency using loop-specific heterogeneity estimates for ADL.

Loop	Ror	seIF	z_value	*p*_value	CI_95	Loop_Heterog_tau2
G-B-E	2.645	2.445	1.082	0.279	(0.00, 7.44)	0.336

B-Western medicine. E-Acupuncture + Herbal medicine + Western medicine. G-Herbal medicine + Western medicine.

**Table 7:** Inverted triangle diagram for ADL.

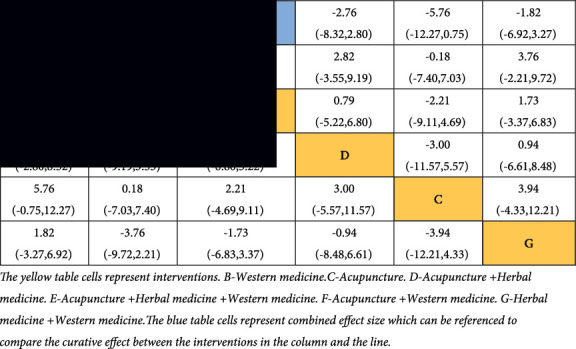

**Table 8:** Adverse events in the included studies.

	Treatment	Time	Symptoms (quantity-percent%)
Ou et al. [32]	Western medicine	—	Nausea, dizziness, and dry mouth (2)
*Zhou* [67]	Western medicine	After 4 weeks of treatment	Nausea, loss of appetite, and diarrhea (3)
	Herbal medicine + acupuncture	—	Diarrhea, loss of appetite and headache (6)
*Li et al.* [11]	Western medicine	—	Dizziness (1) and abdominal distension (1)
	Western medicine + acupuncture + herbal medicine	—	Anorexia (1)
*Wang and Wang* [53]	Western medicine	—	Nausea and vomiting (3); diarrhea (3); dizziness and insomnia (2); and muscle spasm (3)
	Western medicine + acupuncture + herbal medicine	—	Nausea and vomiting (2); diarrhea (5); dizziness and insomnia (4); and muscle spasm (2)
*Zhang* [24]	Western medicine	During the increase of drug dose	6 cases (14.63%)showed adverse drug reactions, including dizziness (3); nausea (1); and decreased appetite (2)
	Western medicine + acupuncture + herbal medicine	—	5 cases (12.20%) had adverse drug reactions, including dizziness(1); vomiting(1); headache(1); and decreased appetite(2)
*Wei et al.* [26]	Western medicine	—	Abnormal liver function and abdominal distension (1–3.03%); abnormal blood routine (3–9.09%); diarrhea (2–6.06%)
	Western medicine + acupuncture	—	Abnormal liver function, abnormal blood routine, abdominal distension (2–6.06%), and diarrhea (4–12.12%)
*Wang and Li* [51]	Western medicine	—	Nausea (1), vomiting (1), diarrhea (2), and cough (1)
	Western medicine + acupuncture	—	Nausea (2), diarrhea (1), and cough (1)
*Zhang et al.* [46]	Western medicine	—	Nausea, vomiting (4); metabolic acidosis (1); diarrhea (3); and pallor (2)
	Herbal medicine + acupuncture	—	Nausea, vomiting(2), and pallor(1)
*Xia et al*. [56]	Western medicine	—	(8–17.39%): diarrhea (2), nausea or vomiting (5), and insomnia (1)
	Western medicine + acupuncture	—	(2–4.35%): Nausea and vomiting (2)
*Zhang et al*. [34]	Western medicine	—	Diarrhea (2), nausea or vomiting (5), and insomnia (1)
	Western medicine + acupuncture	—	Nausea and vomiting (1)
*Tao and Li* [47]	Western medicine	—	(6–13.3%): diarrhea (2), abnormal blood routine (2), abnormal liver function (1), and abdominal distension (1)
	Herbal medicine + acupuncture	—	(5–11.11%): diarrhea (1), abnormal blood routine (1), abnormal liver function (1), and abdominal distension (2)

## Abbreviations

AD: Alzheimer's disease
ADL: Activity of daily living scale
ATD: Alzheimer type dementia
CBM: China Biology Medicine Disc
CI: Credibility interval
CNKI: China National Knowledge Infrastructure
CQVIP: Chongqing VIP Database
DSM-IVR: The Diagnostic And Statistical Manual Of Mental Disorders Revised Fourth Edition
IF: Inconsistency factors
MD: Mean difference
NMA: Network meta-analysis
PICOS: P-Population; I-Intervention; C-Comparison; O-Outcome; S-Study design
PRISMA: Preferred reporting items for systematic reviews and meta-analyses
RCTs: Randomized controlled trials
SD: Standard deviation
SE: Standard error
SUCRA: The surface under the cumulative ranking curve
TCM: Traditional Chinese medicine
